# Crosslinking of Electrospun Fibres from Unsaturated Polyesters by Bis-Triazolinediones (TAD)

**DOI:** 10.3390/polym11111808

**Published:** 2019-11-04

**Authors:** Viviane Chiaradia, Saltuk B. Hanay, Scott D. Kimmins, Débora de Oliveira, Pedro H. H. Araújo, Claudia Sayer, Andreas Heise

**Affiliations:** 1 Department of Chemical Engineering and Food Engineering, Federal University of Santa Catarina (UFSC), Florianópolis, SC 88040-900, Brazil; 2 Department of Chemistry, Royal College of Surgeons in Ireland, 123 St. Stephens Green, Dublin 2, Ireland

**Keywords:** aliphatic polyesters, electrospinning, triazolinediones, fibres

## Abstract

Crosslinking of an unsaturated aliphatic polyester poly(globalide) (PGl) by bistriazolinediones (bisTADs) is reported. First, a monofunctional model compound, phenyl–TAD (PTAD), was tested for PGl functionalisation. ^1^H-NMR showed that PTAD–ene reaction was highly efficient with conversions up to 97%. Subsequently, hexamethylene bisTAD (HM–bisTAD) and methylene diphenyl bisTAD (MDP–bisTAD) were used to crosslink electrospun PGl fibres via one- and two-step approaches. In the one-step approach, PGl fibres were collected in a bisTAD solution for *in situ* crosslinking, which resulted in incomplete crosslinking. In the two-step approach, a light crosslinking of fibres was first achieved in a PGl non-solvent. Subsequent incubation in a fibre swelling bisTAD solution resulted in fully amorphous crosslinked fibres. SEM analysis revealed that the fibres’ morphology was uncompromised by the crosslinking. A significant increase of tensile strength from 0.3 ± 0.08 MPa to 2.7 ± 0.8 MPa and 3.9 ± 0.5 MPa was observed when PGI fibres were crosslinked by HM–bisTAD and MDP–bisTAD, respectively. The reported methodology allows the design of electrospun fibres from biocompatible polyesters and the modulation of their mechanical and thermal properties. It also opens future opportunities for drug delivery applications by selected drug loading.

## 1. Introduction

Aliphatic polyesters are among the most widely used synthetic polymers in biomedical applications, including nanoparticles for drug delivery [1] and scaffolds for tissue regeneration [2,3]. While medium size lactones such as caprolactone and lactide are the most common feedstock monomers [4,5,6], aliphatic polyesters from the ring-opening polymerisation of macrolactones have created some interest as alternative biocompatible polymers [7]. Specifically, unsaturated macrolactones (e.g., globalide) can be used to introduce structural handles for further post-polymerisation modification, a transformation that is very difficult to achieve with conventional lactone derived polyesters. For example, it was demonstrated that hydroxyl and amino groups [8,9], controlled radical initiators [10], poly(ethylene glycol) (PEG) [11], and the drug N-acetylcysteine [12] could be attached to poly(globalide) (PGl) by thiol–ene reactions of the respective functional thiols with unsaturated bonds in the polymer backbone. The unsaturation can further be exploited to crosslink PGl to obtain novel biomaterials for drug delivery systems [6,8,13,14].

Electrospinning is a particularly useful technique to produce porous, high surface area mats of sub-nano to micron size fibres [15,16,17]. It has been exploited to design materials for tissue engineering [18,19], drug delivery applications [20,21,22], as well as supports to immobilise and entrap proteins and enzymes [23,24,25]. Crosslinking of electrospun fibres, either during or post-electrospinning, has recently been proposed as an approach to manipulate the fibre properties while maintaining their structural integrity. Examples include fibres from natural polymers such as diisocyanate crosslinked gelatine [26], hybrid systems, for example, UV-crosslinked collagen/poly(vinyl alcohol) fibres for improved control of salicylic acid release [27], as well as synthetic polymers such as UV-mediated thiol–ene crosslinked oxazoline fibres [28]. We have recently disclosed the first example of thiol–ene crosslinked electrospun fibres from unsaturated PGl polyesters and demonstrated soak loading of drugs in organic solvents without compromising fibre morphology [29]. While these materials offer clear advantages, including their biocompatibility and degradability, the spinning and *in situ* crosslinking of the fibres presented some challenges inherent to the electrospinning process. The latter comprises the extrusion of the formulation containing a solvent, PGl and hexamethylene dithiol through the nozzle followed by passing through a UV light for crosslinking. In this step, all volatile components evaporate, producing a dry crosslinked fibre. Due to the presence of the dithiol, this creates a strong odour, which limits the use of this system in most nonchemistry laboratories. We were thus seeking a different chemical approach that utilises the PGl double bonds and allows efficient fibre crosslinking yet omits the use of dithiols. Triazolinediones (TADs) were identified as suitable alternative crosslinkers. TADs have recently undergone a resurgence in use, mainly for the modification and crosslinking of polymers, due to advances in TAD synthesis and their ability to react in a matter of seconds to minutes at ambient temperatures without the need for any catalyst [30,31]. TADs undergo a number of reactions, the most important ones being electrophilic aromatic substitution, the Diels–Alder reaction and the Alder–ene reaction with highly activated aryl systems, conjugated dienes, and alkenes, respectively [21]. As the double bond is preserved in the Alder–ene reaction, further functionalisation can be undertaken utilising the shifted double bond. TADs have been used to modify unsaturated polymers [32], prepare films [33], produce hydrogels [34,35] and produce crosslinked nanostructures [36]. In one example, a bis-functional TAD was applied to unsaturated fatty acids to produce crosslinked films from vegetable oils [37,38]. Moreover, Heijden and co-workers studied the post-treatment of electrospun styrene–butadiene–styrene (SBS) triblock copolymer fibres by submerging the membranes in TAD solutions in order to improve their mechanical properties [39,40].

Here, for the first time, we utilised the fast and selective TAD chemistry for the modification and crosslinking of unsaturated poly(macrolactone)s. By optimising the reaction conditions, crosslinked electrospun aliphatic polyester fibres were obtained. Through this process, surface crosslinked semi-crystalline fibres as well as fully amorphous fibres with improved mechanical strength and elongation at break were obtained. This methodology offers an avenue to crosslinked fibres from aliphatic polyesters, a class of materials extensively used as biomaterials.

## 2. Materials and Methods

### 2.1. Chemicals

All chemicals were purchased from Sigma-Aldrich unless otherwise noted. Globalide (Gl, 97% purity) was purchased from Symrise, Holzminden, Germany and immobilised lipase B from *Candida antarctica* (Novozym 435) was purchased from Novozymes S/A, Kalundborg, Denmark. Dry ethanol was purchased from Romil, Cambridge, United Kingdom. Hexamethylene bisTAD (HM–bisTAD) and methylene diphenyl bisTAD (MDP–bisTAD) were synthesised as previously reported (see supplementary information for methodology and characterisation, Figures S1–S6) [35].

### 2.2. Synthesis of Poly(globalide) (PGl)

**Figure 1:**
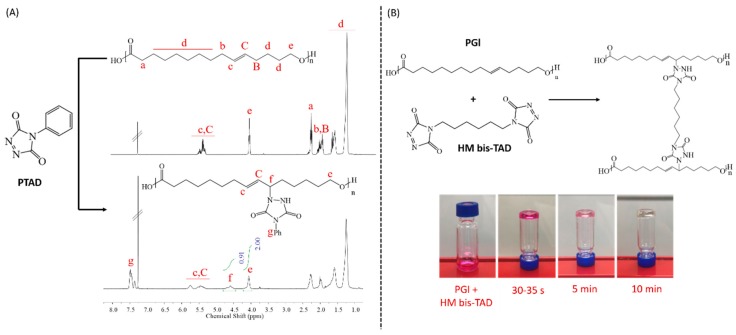
(**A**) Proton nuclear magnetic resonance (^1^H-NMR) spectra of poly(globalide) (PGl), and PGl reacted with phenyl– TAD (PTAD); (**B**) reaction of PGl with hexamethylene bisTAD (HM–bisTAD).

PGl synthesis was conducted in toluene at a globalide: toluene mass ratio of 1:2 (10 g of globalide:20 g of dried toluene) and 6 wt % of Novozym 435 in relation to monomer. The reaction proceeded for 4 h at 60 °C. Then, dichloromethane (DCM) was added, and the final solution was filtered and precipitated in cold methanol. The precipitate was dried under vacuum at room temperature to result in a polymer with a number average molecular weight (*M*_n_) of 20,000 g/mol (Đ: 3.5, Figure S7). Yield: 80%. ^1^H-NMR with peak assignments depicted in Figure 1A.

### 2.3. Modification of PGl with PTAD

One hundred milligrams of PGl was dissolved in 0.5 mL chloroform. PTAD (TAD: ene molar ratio ca. 1:1) measuring 72.7 mg (0.415 mmol) was dissolved in acetonitrile and then added to the PGl solution. The reaction was allowed to proceed for 3 h at room temperature. After PTAD addition, the colour changed from red to maroon, brown and finally pale yellow (Figure S8). Finally, the solution was precipitated in acetonitrile and the obtained polymer was dried under vacuum for further characterisation. Yield: 81%. ^1^H-NMR with peak assignments is depicted in Figure 1A.

### 2.4. Crosslinking of PGl with HM–bisTAD

For materials imaged in Figure 1B, 3 mg of PGl (0.0125 mmol) was dissolved in 0.1 mL of chloroform. Then, 4.36 mg (0.0155 mmol) of HM–bisTAD was dissolved in 0.1 mL acetonitrile/chloroform mixture (60/40). Then, the HM–bisTAD solution was added to the PGl solution, and the vials were monitored until the disappearance of the pink colour and gel formation.

### 2.5. Electrospinning of PGl and In-Situ Crosslinking by bisTADs

The electrospinning was performed using a Spraybase electrospinning machine with a stationary collector and a nozzle diameter of 1.02 mm (18 G) based on a protocol described previously by our group at room temperature without specific humidity control [29]. PGl concentration was kept at 30 wt % (in DCM). Voltage, flow rate and distance from tip to collector were set at 12 kV, 100 μL/min and 15 cm, respectively. PGl fibres were collected in a 10 wt % bisTAD (HM–bisTAD or MDP–bisTAD) solution in acetonitrile. Then, the crosslinked fibres were rinsed in acetonitrile and dried under vacuum prior to characterisation.

### 2.6. Post-Crosslinking of PGl Fibres by bisTADs

PGl fibres were obtained using a Spraybase electrospinning machine applying the same conditions described in Section 2.5, with the only exception being that the fibres were collected on a Teflon plate covered with aluminium foil. The electrospun mats were cut in a dog-bone shape and submerged in a TAD solution (HM–bisTAD or MDP–bisTAD) in acetonitrile using a molar ratio TAD: ene of circa 1:2 and TAD concentration of 0.018 mol/L. After 24 h, the fibres were washed with an excess of acetonitrile to remove any unreacted TAD and dried under vacuum. In order to achieve further crosslinking, the partially crosslinked fibres were submerged in a second TAD solution at the same concentration as for the first crosslinking but using a mixture of chloroform and acetonitrile as solvents (1:1).

### 2.7. Methods

^1^H-NMR spectra were recorded using a Bruker spectrometer at 400 MHz. Chemical shifts were reported in parts per million (ppm) using tetramethylsilane (TMS)-0.01% (*v/v*) (δ = 0.00 ppm) as internal standard. The samples were solubilised in CDCl_3_ (δ = 7.26 ppm). Thermal analysis of the modified polymers was conducted using a TA Instruments Q200 DSC, Newcastle, United Kingdom, using approximately 9.0 mg of dried purified polymer. Temperature profiles from −10 to 120 °C with a heating and cooling rate of 10 °C/min were applied under nitrogen atmosphere. The melting temperatures were determined by the second heating. The morphology was verified by scanning electron microscopy (SEM) with a Zeiss Ultra Plus SEM instrument, Oberkochen, Germany (Gemini column). Samples were placed on conductive carbon stubs and coated with Pt/Pd. An accelerating voltage of 2 kV was used for all samples. Fibre size distribution was accessed by selecting randomly at least 100 fibres for each image using the software ImageJ, Bethesda, MD, USA, and reported as an average. Mechanical properties as tensile strength and elongation at break were measured using a Zwick/Roell model Z2 machine, Herefordshire, United Kingdom, equipped with a 50 N load cell. The electrospun mats were cut in a dog-bone shape (63.6 mm in length and ca. 1.2 mm in thickness) and 5 measurements of each sample were performed.

## 3. Results and Discussion

### 3.1. TAD Modification and Crosslinking of PGl

Macrolactones can readily be polymerised by enzymatic ring-opening polymerisation [7,41,42,43]. Here, PGl was obtained by the *Candida antarctica* Lipase B (CALB) catalysed polymerisation of Gl at a molecular weight of 20,000 g/mol (Đ: 3.5). In order to demonstrate the feasibility of the PGl modification via a triazolinedione reaction, commercial 4–phenyl–1,2,4–triazoline–3,5-dione (PTAD) was used as a monofunctional model compound. The reaction between PGl and PTAD was investigated in THF using a fixed amount of the reactants (molar ratio PGl–ene to PTAD circa 1:1). The reaction could be monitored visually by the disappearance of the red PTAD colour (Figure S8, ESI). After precipitation, the ^1^H-NMR spectrum of the polymer revealed aromatic protons ascribed to the conjugated PTAD (g in Figure 1A) as well as a new signal centred at 4.6 ppm assigned to the newly formed C–N bond due to the urazole introduction (f in Figure 1A). Comparison of this signal with the signal at 4.1 ppm (e in Figure 1A) revealed a double bond conversion of >90%. Notably, PGl is a semicrystalline polymer, but after PTAD modification, a transparent pale yellow glass-like polymer was obtained. Thermal analysis (Figure S9) confirmed the reduction in polymer crystallinity in agreement with previous reports on PGl double bond modification [9]. In the next experiment, the crosslinking of PGl was attempted by adding a chloroform/acetonitrile solution of bisTAD to a solution of PGl in chloroform (Figure 1B). Hexamethylene bisTAD (HM–bisTAD) and methylene diphenyl bisTAD (MDP–bisTAD) were selected, as both have previously been used in crosslinking reactions [35]. Irrespective of the selected bisTAD, the final mixture became viscous within 30–35 seconds, and gelation was observed (Figure 1B). After 10 minutes, the pink colour completely disappeared, indicating a successful TAD crosslinking of the PGl. A swelling test in dichloromethane (DCM) revealed that the obtained organogels could absorb about 16 times their own weight.

### 3.2. Electrospinning of PGl and TAD Crosslinking

**Figure 2:**
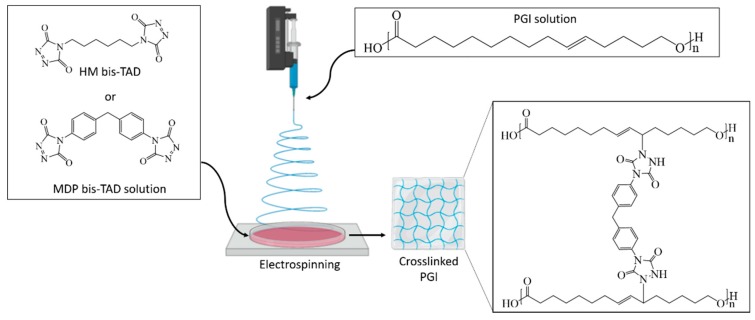
Electrospinning of PGl fibres directly into a bisTAD containing collector solution; note that only the MDP-bisTAD crosslinked product is depicted.

**Figure 3:**
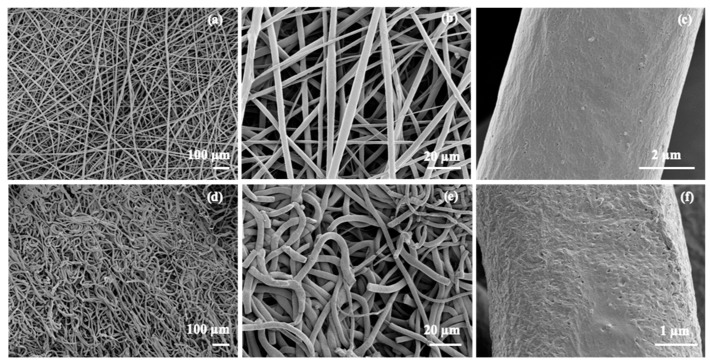
SEM images of electrospun PGl (**a**–**c**) and PGl collected in a solution of MDP–bisTAD (**d**–**f**).

The crosslinking experiments highlighted that reaction between the bisTADs and the PGl occurs too fast to mix both components prior to the electrospinning. To overcome this problem, we devised a method in which the PGl fibres were spun first and subsequently crosslinked by incubation in a bisTAD solution for post-spinning crosslinking (Figure 2). Therefore, the PGl was solubilised in DCM, and the electrospinning parameters were set according to a literature protocol [29]. In the first instance, the fibres were collected directly in a mixture of HM– or MDP–bisTAD in acetonitrile in order to allow *in situ* crosslinking in the collector. Acetonitrile was selected as a nonsolvent for PGl to avoid fibre dissolution. As observed in Figure 3, homogeneous PGl fibres of around 10 µm were obtained (fibre size distribution, Figure S10). While diameters are comparable to non-crosslinked fibres, SEM images also reveal notable differences between crosslinked and non-crosslinked fibres. Overall, non-crosslinked fibres appear more homogeneous (Figure 3a–c), while *in situ* crosslinked fibres display a more wrinkled surface morphology (Figure 3d–f). However, large parts of the fibres could be dissolved, indicating incomplete crosslinking with bisTADs in the collector, thereby limiting the feasibility of this approach.

**Figure 4:**
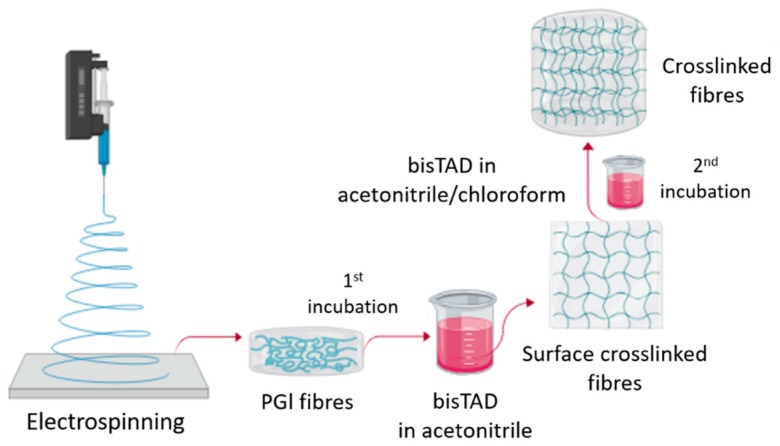
Two-step crosslinking procedure to obtain PGl fibres with different degrees of crosslinking.

We hypothesised that the first approach produced only inhomogeneously crosslinked fibres due to the slow diffusion of bisTAD into the material. However, the fact that the crosslinking can be controlled by the diffusion of the bisTAD into the fibres opens interesting opportunities. For example, it should allow us to use the degree of crosslinking to manipulate the fibre’s mechanical properties. To further investigate this, dry fibres were first collected and subjected to a two-step crosslinking process as depicted in Figure 4. In the first step, the fibres were incubated in bisTAD solution in the non-solvent acetonitrile to achieve lightly crosslinked fibres. It was rationalised that this would result in crosslinking of the PGl present on the fibre surfaces. Full crosslinking was then achieved by a second incubation in a bisTAD solution containing a solvent mixture of acetonitrile and chloroform. The latter is a good solvent for PGl and was envisaged to facilitate fibre swelling, enabling bulk diffusion and crosslinking by bisTAD. No dissolution of the fibres in this second step was observed; instead, solvent swelling of the fibres occurred, supporting the notion of surface crosslinking in the first step. The swollen fibres were left to react with bisTAD in the second solution for 24 h to ensure enough time to complete the reaction. The gel content was accessed by incubation of the fibres in chloroform to remove any unreacted PGl. After incubation, the fibres were dried to constant weight and gel contents of around 80% and 95% were determined after the first and second crosslinking, respectively.

**Figure 5:**
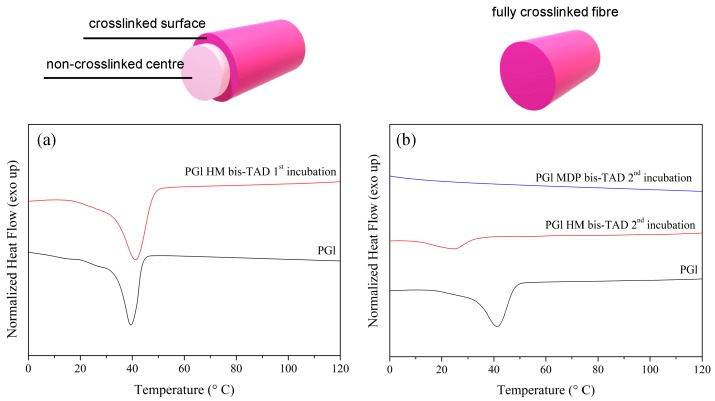
Differential scanning calorimetry thermograms (second heating curve) of (**a**) first incubation of PGl fibres and (**b**) second incubation of partial crosslinked PGl fibres in HM–bisTAD and MDP–bisTAD.

**Figure 6:**
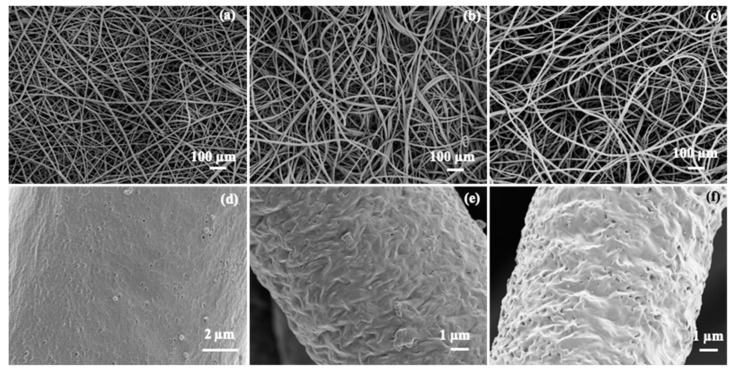
SEM images of plain PGl (**a**,**d**); crosslinked PGl/HM–bisTAD (**b**,**e**) and crosslinked PGl MDP–bisTAD (**c**,**f**).

Differential scanning calorimetry (DSC) analysis showed a defined melting temperature (*T*_m_) of around 40 °C after the first crosslinking in agreement with the semicrystalline structure of PGl (Figure 5a). These results are in agreement with partly or lightly crosslinked fibres, presumably at the fibre–solvent interface, thereby sealing the fibre surfaces, while inner parts of the fibres remained un-crosslinked. After the second crosslinking, DSC analysis (Figure 5a) confirmed a significant reduction of crystallinity for the HM–bisTAD treated samples and completely amorphous polymer fibres for the MDP–bisTAD treated samples (Figure 5b). SEM images in Figure 6 highlight that the fibre morphology is retained after the second crosslinking procedure and diameters remain around 10–12 µm for crosslinked fibres irrespective of the bisTAD used. These values are comparable to the untreated PGl fibres of around 10 µm (fibre diameter distributions in Figure S12).

**Figure 7:**
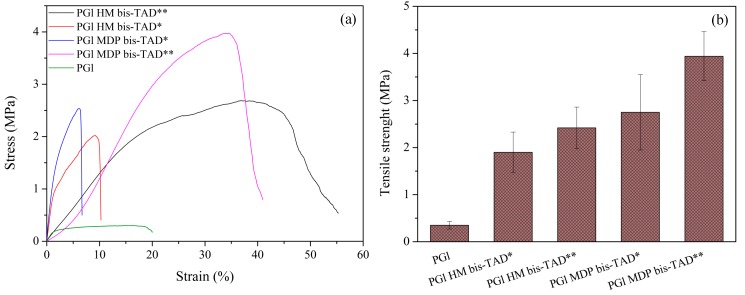
(**a**) Stress strain curves; (**b**) and tensile strength of PGl, PGl HM–bisTAD* (first incubation), PGl HM–bisTAD** (second incubation), PGl MDP–bisTAD* (first incubation) and PGl MDP–bisTAD (second incubation) fibres.

After the two-step crosslinking, an improvement in the fibres’ mechanical properties was observed (Figure 7). While non-crosslinked PGl fibres presented a tensile strength of 0.3 ± 0.08 MPa, crosslinking with HM–bisTAD and MDP–bisTAD increased values to around 1.9 ± 0.43 and 2.4 ± 0.44 MPa after first incubation in TAD solution and 2.7 ± 0.8 and 3.9 ± 0.5 MPa after second bisTAD incubation, respectively, which corresponds to 600%, 800%, 900%, and 1300% higher values than those of untreated fibres. This represents a significant improvement over fibres obtained via electrospinning/*in situ* UV-initiated thiol–ene crosslinking with only a 194% increase in the tensile strength when compared with non-crosslinked PGl [29]. After the second incubation, the fibres also showed increased elongation at break when compared with the first crosslink (from 8.5% to 45% and from 7.5% to 31% when HM–bisTAD and MDP–bisTAD were used, respectively).

## 4. Conclusions

We devised a novel approach to crosslinked polyester fibres combining electrospinning and robust, fast and efficient TAD chemistry. By selecting the correct solvent/TAD combination for the incubation of fibres, surface crosslinked fibres with a semicrystalline core and fully crosslinked amorphous fibres with enhanced mechanical properties were obtained. The presented approach opens possibilities beyond the modulation of mechanical properties, for example, selective loading of drugs into the crosslinked fibres for drug delivery applications.

## Supplementary Materials

The following are available online at https://www.mdpi.com/2073-4360/11/11/1808/s1, Experimental procedures, Figure S1: ^1^H-NMR spectrum of hexamethylene bishydrazine carboxylate in DMSO-d6., Figure S2: ^1^H-NMR spectrum of hexamethylene bisurazole in DMSO-d6. Figure S3: ^1^H-NMR spectrum of hexamethylene bisTAD in DMSO-d6. Figure S4: ^1^H-NMR of methylene diphenyl bishydrazine carboxylate in DMSO-d6. Figure S5: ^1^H-NMR spectrum of methylene diphenyl bisurazole in DMSO-d6. Figure S6: ^1^H-NMR spectrum of methylene diphenyl bisTAD in DMSO-d6. Figure S7: GPC trace of poly(globalide) homopolymer. Figure S8: Reaction medium after initial mixing, 3 hours and after overnight reaction of PTAD and PGl using THF as solvent. Figure S9: Differential scanning calorimetry thermograms (second heating curve) before and after modification of PGl with PTAD. Figure S10: Fibre size distribution of PGl fibres (a) and crosslinked fibres with MDP-bisTAD after electrospinning of PGl and fibre collection in bisTAD solution (b). Figure S11: Additional SEM images of crosslinked PGl/HM-bisTAD (a,c) and PGl/MDP-bisTAD after swelling in dichloromethane. Figure S12: Fibre size distribution of crosslinked PGl/HM-bisTAD fibres (a) and crosslinked PGl/MDP-bisTAD (b) after second incubation in TAD solutions. Figure S13: Photographic images of non-crosslinked PGl fibres (a), fully crosslinked PGl/HM-bisTAD fibres (b) and fully crosslinked PGl/MDP-bisTAD fibres (c).

## References

[1] Zheng L., Zhang X., Wang Y., Liu F., Peng J., Zhao X., Yang H., Ma L., Wang B., Chang C. (2018). Fabrication of acidic pH-cleavable polymer for anticancer drug delivery using a dual functional monomer. Biomacromolecules.

[2] Oh G.W., Ko S.C., Je J.Y., Kim Y.M., Oh J.H., Jung W.K. (2016). Fabrication, characterization and determination of biological activities of poly(ε-caprolactone)/chitosan-caffeic acid composite fibrous mat for wound dressing application. Int. J. Biol. Macromol..

[3] Pawar M.D., Rathna G.V.N., Agrawal S., Kuchekar B.S. (2015). Bioactive thermoresponsive polyblend nanofiber formulations for wound healing. Mater. Sci. Eng. C.

[4] Labet M., Thielemans W. (2009). Synthesis of polycaprolactone: A review. Chem. Soc. Rev..

[5] Danhier F., Ansorena E., Silva J.M., Coco R., Le Breton A., Préat V. (2012). PLGA-based nanoparticles: An overview of biomedical applications. J. Control. Release.

[6] Malikmammadov E., Tanir T.E., Kiziltay A., Hasirci V., Hasirci N. (2018). PCL and PCL-based materials in biomedical applications. J. Biomat. Sci. Polym. Ed..

[7] Wilson J.A., Ates Z., Pflughaupt R.L., Dove A.P., Heise A. (2019). Polymers from macrolactones: From pheromones to functional materials. Prog. Polym. Sci..

[8] Ates Z., Heise A. (2014). Functional films from unsaturated poly(macrolactones) by thiol–ene cross-linking and functionalization. Polym. Chem..

[9] Ates Z., Thornton D., Heise A. (2011). Side-chain functionalisation of unsaturated polyesters from ring-opening polymerisation of macrolactones by thiol – ene click chemistry. Polym. Chem..

[10] Ates Z., Audouin F., Harrington A., O’Connor B., Heise A. (2014). Functional brush-decorated poly(globalide) films by ARGET-ATRP for bioconjugation. Macromol. Biosci..

[11] Savin C.L., Peptu C., Kroneková Z., Sedlačík M., Mrlik M., Sasinková V., Peptu C.A., Popa M., Mosnáček J. (2018). Polyglobalide-based porous networks containing poly(ethylene glycol) structures prepared by photoinitiated thiol–ene coupling. Biomacromolecules.

[12] Guindani C., Dozoretz P., Araújo P.H.H., Ferreira S.R.S., De Oliveira D. (2019). N-acetylcysteine side-chain functionalization of poly(globalide-co-caprolactone) through thiol-ene reaction. Mater. Sci. Eng. C.

[13] Claudino M., Van Der Meulen I., Trey S., Jonsson M., Heise A., Johansson M. (2012). Photoinduced thiol-ene crosslinking of globalideε-caprolactone copolymers: Curing performance and resulting thermoset properties. J. Polym. Sci. Part A Polym. Chem..

[14] Van Der Meulen I., Li Y., Deumens R., Joosten E.J., Koning C.E., Heise A. (2011). Copolymers from unsaturated macrolactones: Toward the design of cross-linked biodegradable polyesters. Biomacromolecules.

[15] Jian S., Zhu J., Jiang S.H., Chen S.L., Fang H., Song Y., Duan G.G., Zhange Y.F., Hou H.Q. (2018). Nanofibers with diameter below one nanometer from electrospinning. RSC Adv..

[16] Li D., Xia Y. (2004). Electrospinning of Nanofibers: Reinventing the Wheel?. Adv. Mater..

[17] Bhardwaj N., Kundu S.C. (2010). Electrospinning: A fascinating fiber fabrication technique. Biotechnol. Adv..

[18] Sill T.J., von Recum H.A. (2008). Electrospinning: Applications in drug delivery and tissue engineering. Biomaterials.

[19] Jun I., Han H.S., Edwards J.R., Jeon H. (2018). Electrospun fibrous scaffolds for tissue engineering: Viewpoints on architecture and fabrication. Int. J. Mol. Sci..

[20] Duan G., Bagheri A.R., Jiang S., Golenser J., Agarwal S., Greiner A. (2017). Exploration of macroporous polymeric sponges as drug carriers. Biomacromolecules.

[21] Torres-Martinez E.J., Bravo J.M.C., Medina A.S., González G.L.P., Gómez L.J.V. (2018). A summary of electrospun nanofibers as drug delivery system: Drugs loaded and biopolymers used as matrices. Curr. Drug Deliv..

[22] Hu X.L., Liu S., Zhou G.Y., Huang Y.B., Xie Z.G., Jing X.B. (2014). Electrospinning of polymeric nanofibers for drug delivery applications. J. Control. Release.

[23] Jia H., Zhu G., Vugrinovich B., Kataphinan W., Reneker D.H., Wang P. (2002). Enzyme-carrying polymeric nanofibers prepared via electrospinning for use as unique biocatalysts. Biotechnol. Prog..

[24] Zeng J., Aigner A., Czubayko F., Kissel T., Wendorff J.H., Greiner A. (2005). Poly(vinyl alcohol) nanofibers by electrospinning as a protein delivery system and the retardation of enzyme release by additional polymer coatings. Biomacromolecules.

[25] Kalaoglu-Altan O.I., Sanyal R., Sanyal A. (2015). “Clickable” polymeric nanofibers through hydrophilic–hydrophobic balance: Fabrication of robust biomolecular immobilization platforms. Biomacromolecules.

[26] Kishan A.P., Nezarati R.M., Radzicki C.M., Renfro A.L., Robinson J.L., Whitely M.E., Cosgriff-Hernandez E.M. (2015). *In situ* crosslinking of electrospun gelatin for improved fiber morphology retention and tunable degradation. J. Mater. Chem. B.

[27] Zhang X., Tang K., Zheng X.J. (2016). Electrospinning and crosslinking of COL/PVA nanofiber-microsphere containing salicylic acid for drug delivery. Bionic Eng..

[28] Kalaoglu-Altan O.I., Verbraeken B., Lava K., Gevrek T.N., Sanyal R., Dargaville T., De Clerck K., Hoogenboom R., Sanyal A. (2016). Multireactive poly(2-oxazoline) nanofibers through electrospinning with crosslinking on the fly. ACS Macro Lett..

[29] de Oliveira F.C.S., Olvera D., Sawkins M.J., Cryan S.-A., Kimmins S.D., da Silva T.E., Kelly D.J., Duffy G.P., Kearney C., Heise A. (2017). Direct UV-Triggered thiol–ene cross-linking of electrospun polyester fibers from unsaturated poly(macrolactone)s and their drug loading by solvent swelling. Biomacromolecules.

[30] Butler G.B. (1980). Triazolinedione Modified Polydienes. Ind. Eng. Chem. Prod. Res. Dev..

[31] De Bruycker K., Billiet S., Houck H.A., Chattopadhyay S., Winne J.M., Du Prez F.E. (2016). Triazolinediones as highly enabling synthetic tools. Chem. Rev..

[32] Vandewalle S., Van De Walle M., Chattopadhyay S., Du Prez F. (2018). Polycaprolactone-b-poly(N-isopropylacrylamide) nanoparticles: Synthesis and temperature induced coacervation behavior. E. Eur. Polym. J..

[33] Vlaminck L., De Bruycker K., Turunc O., Du Prez F.E. (2016). ADMET and TAD chemistry: A sustainable alliance. Polym. Chem..

[34] Hanay S.B., O’Dwyer J., Kimmins S.D., De Oliveira F.C.S., Haugh M.G., O’Brien F.J., Cryan S.-A., Heise A. (2018). Facile approach to covalent copolypeptide hydrogels and hybrid organohydrogels. ACS Macro Lett..

[35] Hanay S.B., Ritzen B., Brougham D., Dias A.A., Heise A. (2017). Exploring tyrosine-triazolinedione (TAD) reactions for the selective conjugation and cross-linking of N-Carboxyanhydride (NCA) derived synthetic copolypeptides. Macromol. Biosci..

[36] Brannigan R.P., Kimmins S.D., Bobbi E., Caulfield S., Heise A. (2019). Synthesis of novel bis-triazolinedione crosslinked amphiphilic polypept(o)ide nanostructures. Macromol. Chem. Phys..

[37] Türünç O., Billiet S., De Bruycker K., Ouardad S., Winne J., Du Prez F.E. (2015). From plant oils to plant foils: Straightforward functionalization and crosslinking of natural plant oils with triazolinediones. Eur. Polym. J..

[38] Chattopadhyay S., Du Prez F.E. (2016). Simple design of chemically crosslinked plant oil nanoparticles by triazolinedione-ene chemistry. Eur. Polym. J..

[39] Van Der Heijden S., De Bruycker K., Simal R., Du Prez F., De Clerck K. (2015). Use of triazolinedione click chemistry for tuning the mechanical properties of electrospun SBS-fibers. Macromolecules.

[40] Van Der Heijden S., Daelemans L., De Bruycker K., Simal R., De Baere I., Van Paepegem W., Rahier H., De Clerck K. (2017). Novel composite materials with tunable delamination resistance using functionalizable electrospun SBS fibers. Compos. Struct..

[41] Polloni A.E., Chiaradia V., Figura E.M., de Paoli J.P., de Oliveira D., de Oliveira J.V., de Araujo P.H.H., Sayer C. (2018). Polyesters from Macrolactones Using Commercial Lipase NS 88011 and Novozym 435 as Biocatalysts. Appl. Biochem. Biotechnol..

[42] Kumar A., Kalra B., Dekhterman A., Gross R.A. (2000). Efficient ring-opening polymerization and copolymerization of ε-Caprolactone and ω-Pentadecalactone catalyzed by *Candida antartica* lipase B. Macromolecules.

[43] de Geus M., Van Der Meulen I., Goderis B., van Hecke K., Dorschu M., van der Werff H., Koning C.E., Heise A. (2010). Performance polymers from renewable monomers: High molecular weight poly(pentadecalactone) for fiber applications. Polym. Chem..

