# Supplementary material for: Crosslinking of Electrospun Fibres from Unsaturated Polyesters by Bis-Triazolinediones (TAD)
Source: Polymers (Basel). 2019 Nov 4;11(11):1808. doi: 10.3390/polym11111808 (PMC6918174; doi:10.3390/polym11111808)
Supplement: Supplementary file 1 [file polymers-11-01808-s001.pdf]

Crosslinking of electrospun fibres from unsaturated polyesters by bis-triazolinediones (TAD)

Viviane Chiaradia [1], Saltuk B. Hanay [2], Scott D. Kimmins [2], Débora de Oliveira [1], Pedro H. H. Araújo [1], Claudia Sayer [1] and Andreas Heise [2,*]

[1] Department of Chemical Engineering and Food Engineering, Federal University of Santa Catarina (UFSC), Florianópolis, SC 88040-900, Brazil.;
[2] Department of Chemistry, Royal College of Surgeons in Ireland, Dublin 2, Ireland.;

*Correspondence: andreasheise@rcsi.ie

1 Synthesis of Hexamethylene bis-TAD synthesis

Step 1 (Hexamethylene bishydrazine carboxylate)
Ethyl carbazate (10.4 g) was dissolved in 200 mL of anhydrous tetrahydrofuran (THF) under an inert atmosphere in an ice bath. Then, hexamethylene diisocyanate (8 mL) was added dropwise for 10 minutes while stirring. After addition, the reaction was brought to room temperature and mixed for 2 hours. Then the suspension was filtrated, and the product was washed with THF and dried overnight under vacuum to give a pure product with 95 % yield (Figure S1).

Step 2 (Hexamethylene bisurazole)
The product obtained in step 1 (5 g) and potassium carbonate (7.5 g) were suspended in 300 mL of pure ethanol under nitrogen atmosphere. The mixture was allowed to react for 2 days in reflux. After 2 days, ethanol was evaporated, and the crude product was dissolved in a small amount of distilled water. Then, the pH was adjusted to 1.5 by the dropwise addition of concentrated hydrochloric acid (HCl) at 0 ºC. The precipitated fraction was washed with distilled water and dried under vacuum to give a pure product with 87 % yield (Figure S2).

Step 3 (Hexamethylene bis-TAD)
5g of bis-urazole obtained in step 2 was added to a 500 mL round bottom flask and suspended in 250 mL of dichloromethane (DCM) under a nitrogen atmosphere. Silica nitric acid (15g) was prepared following a previous method[1,2] and added slowly over 15 minutes to the flask and then mixed for a further 2 hours. After, the mixture was filtered, dried over anhydrous magnesium sulfate ($MgSO_4$) and the DCM was evaporated to obtain the final product with 62 % yield (Figure S3).

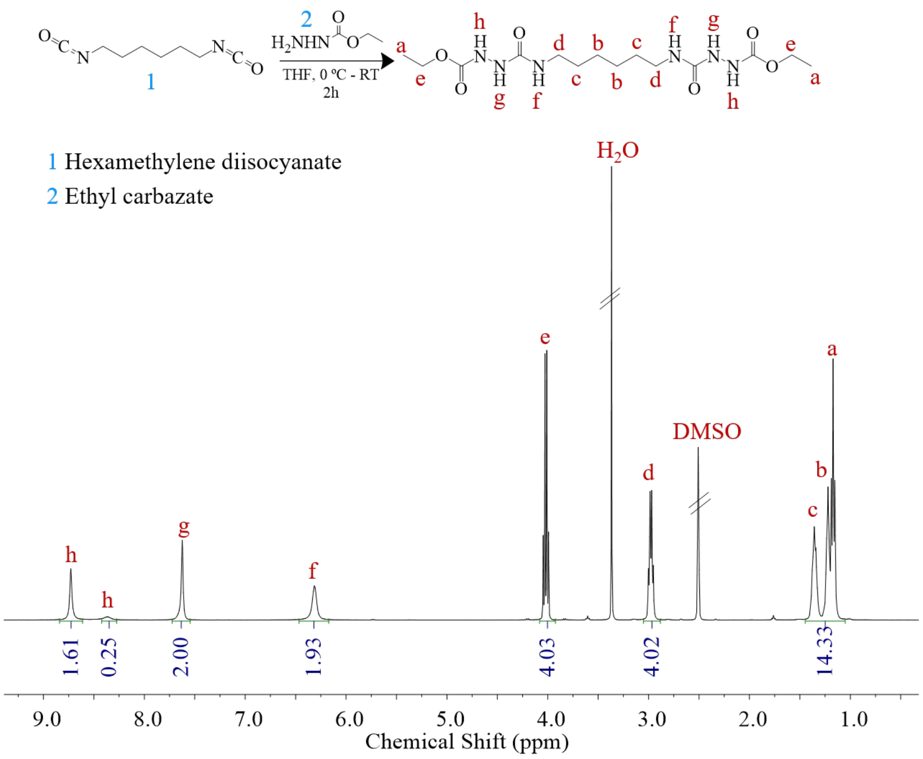

Figure S1: ^{1}H-NMR spectrum of hexamethylene bishydrazine carboxylate in DMSO-d6.

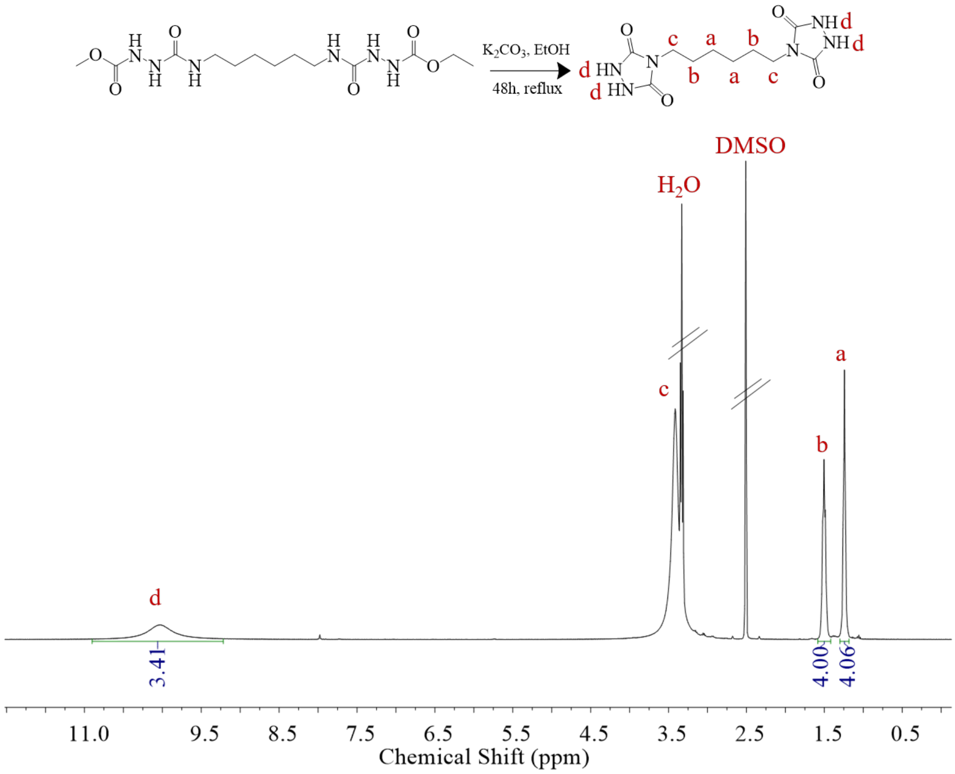

Figure S2: ^{1}H-NMR spectrum of hexamethylene bisurazole in DMSO-d6.

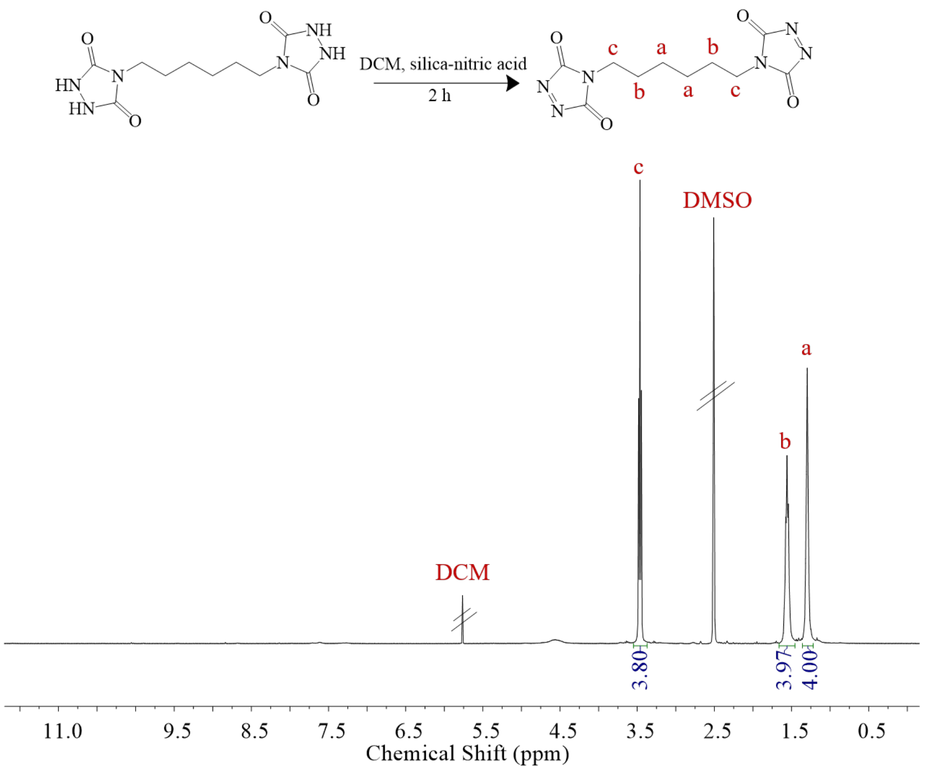

Figure S3: ^{1}H-NMR spectrum of hexamethylene bisTAD in DMSO-d6.

<u>**2 Synthesis of methylene diphenyl bis-TAD synthesis (MDP-bis TAD)**</u>

Step 1 (Methylene diphenyl bishydrazine carboxylate)

Ethyl carbazate (5 g) was dissolved in 30 mL of anhydrous THF under inert atmosphere in an ice bath. Then, methylenediphenyl diisocyanate (6 g) was added to 30 mL of anhydrous THF and added dropwise for 10 minutes while stirring at 0 ºC. After addition, the reaction proceeded at room temperature during 1 h. The final mixture was filtered, and the product was washed with THF and dried overnight under vacuum to give a pure product with 100 % yield (Figure S4).

Step 2 (Methylene diphenyl bisurazole)

The product obtained in step 1 (5 g) and a 4 N solution of potassium hydroxide (KOH) (25 mL) were added in a flask and refluxed for 2 h. The suspension was cooled down and the pH was adjusted to 1.5 by the dropwise addition of concentrated HCl at 0 ºC. After filtration, the precipitated fraction was washed with distilled water and dried to give a pure product with 77 % yield (Figure S5).

Step 3 (Methylenediphenyl bis-TAD)

The oxidation of the urazole (from step 2) was conducted as described before for hexamethylene bis-TAD (Figure S6).

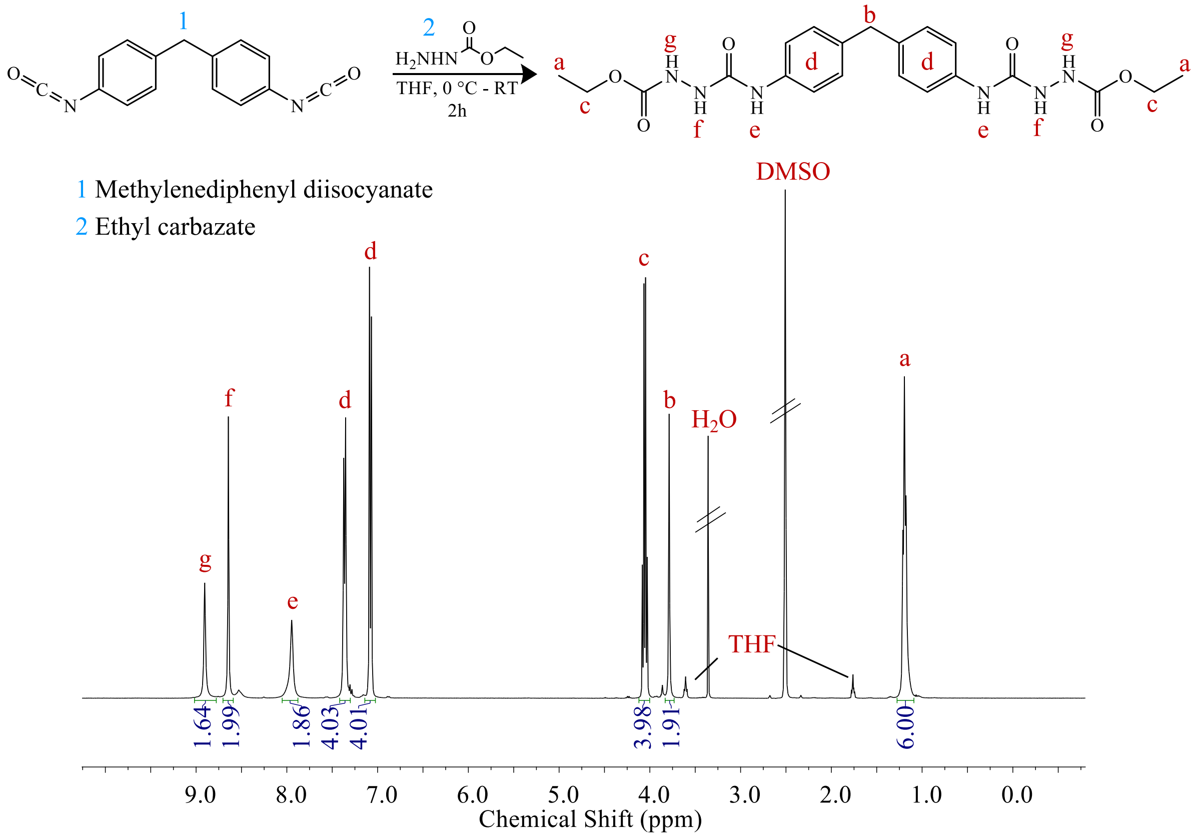

Figure S4: ^{1}H-NMR of methylene diphenyl bishydrazine carboxylate in DMSO-d6.

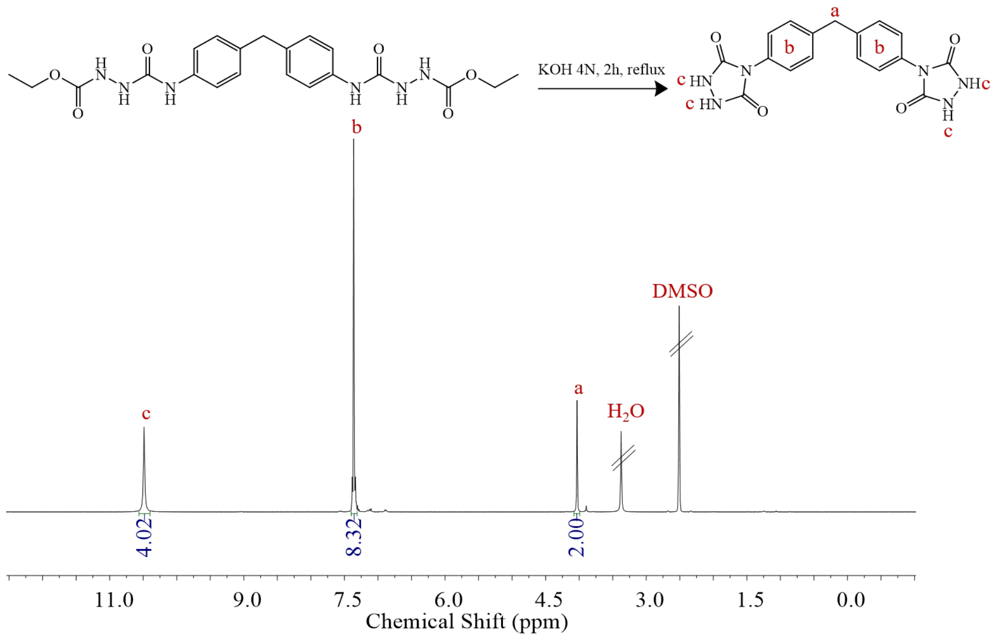

Figure S5: ^{1}H-NMR spectrum of methylene diphenyl bisurazole in DMSO-d6.

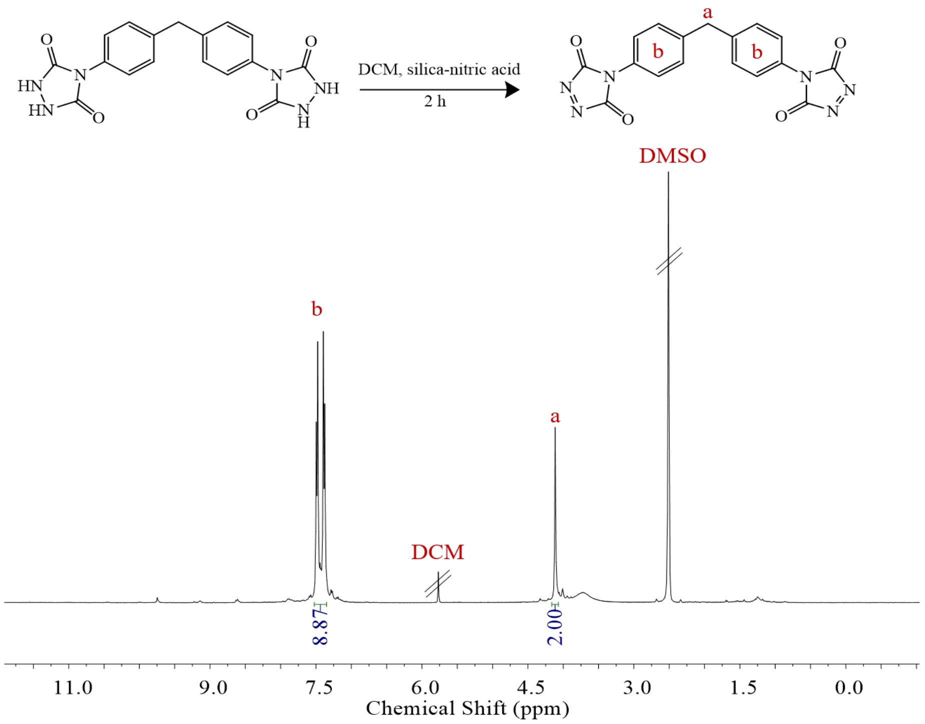

Figure S6: ^{1}H-NMR spectrum of methylene diphenyl bisTAD in DMSO-d6.

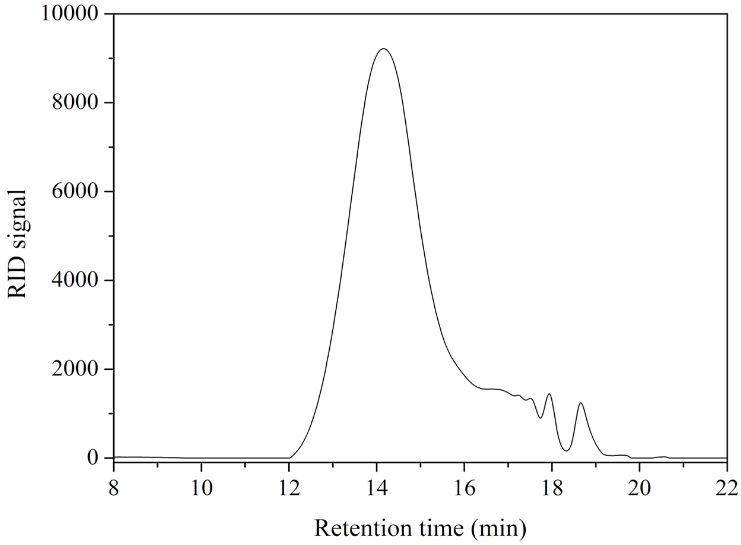

Figure S7: GPC trace of poly(globalide) homopolymer. PGl homopolymer: M_n = 20,000 g mol^{-1}; M_w = 70,000 g mol^{-1}

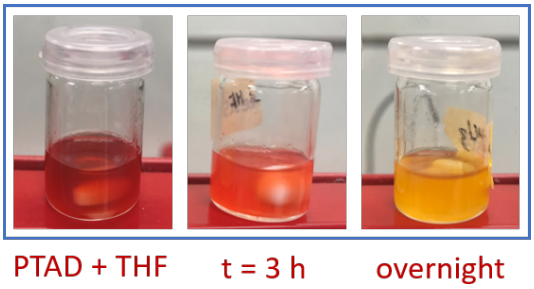

Figure S8: Reaction medium after initial mixing, 3 hours and after overnight reaction of PTAD and PGl using THF as solvent.

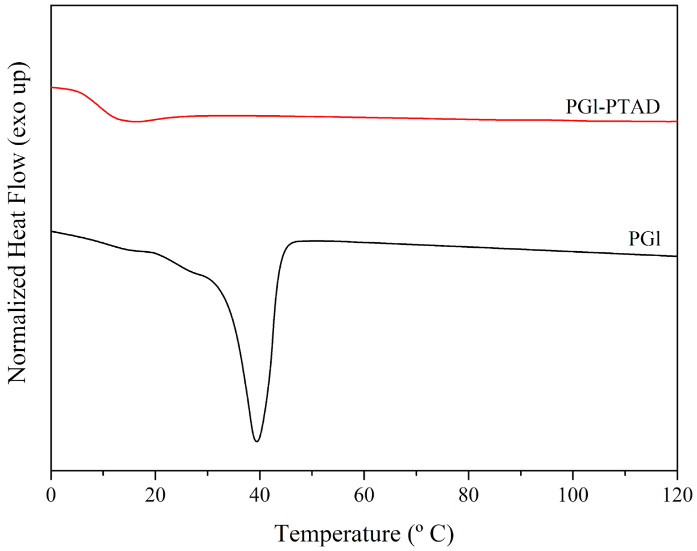

Figure S9: Differential scanning calorimetry thermograms (second heating curve) before and after modification of PGl with PTAD.

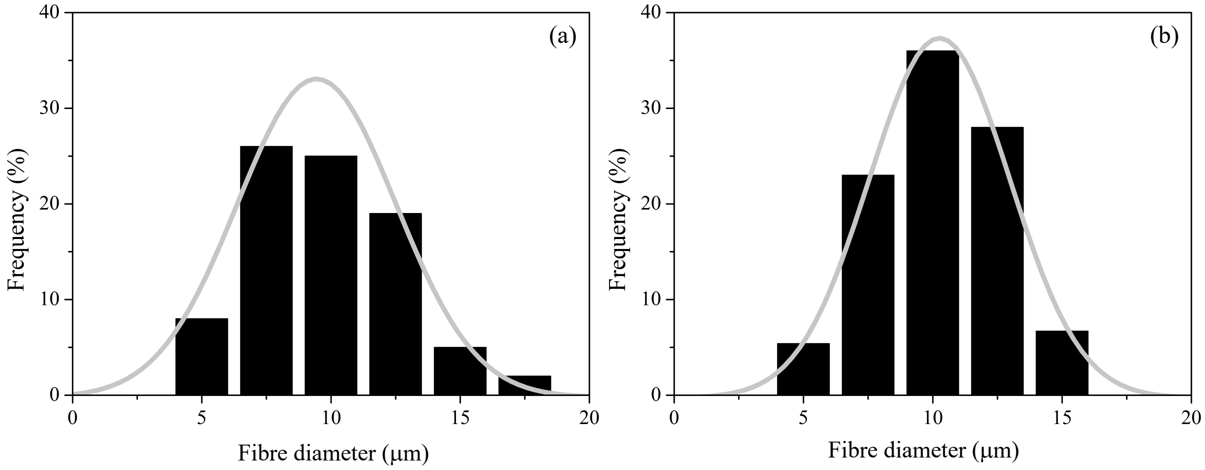

Figure S10: Fibre size distribution of PGl fibres (a) and crosslinked fibres with MDP-bisTAD after electrospinning of PGl and fibre collection in bisTAD solution (b).

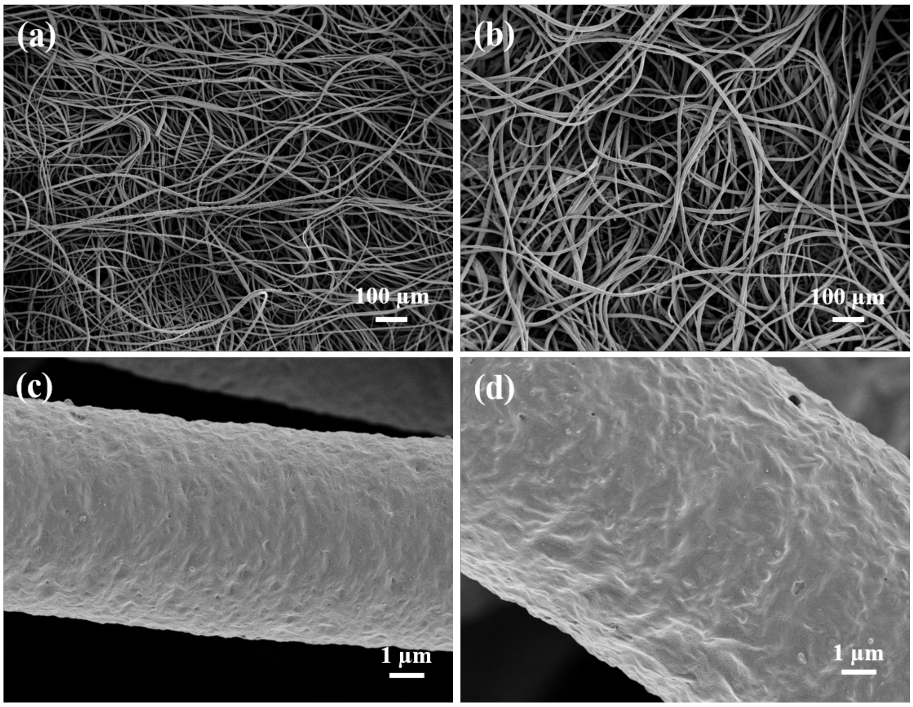

Figure S11: Additional SEM images of crosslinked PGl/HM-bisTAD (a,c) and PGl/MDP-bisTAD after swelling in dichloromethane.

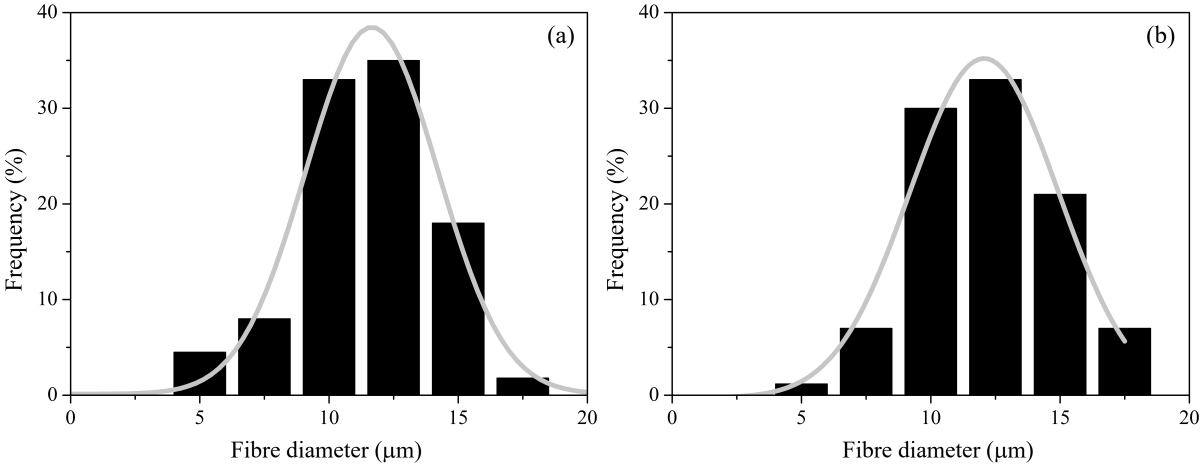

Figure S12: Fibre size distribution of crosslinked PGl/HM-bisTAD fibres (a) and crosslinked PGl/MDP-bisTAD (b) after second incubation in TAD solutions.

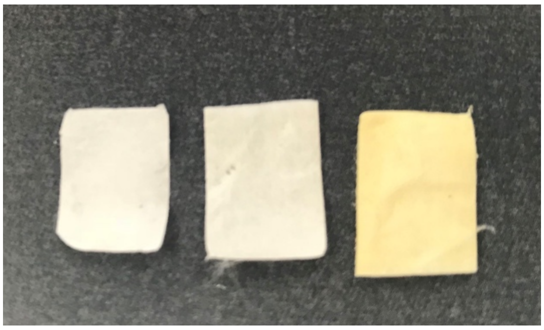

Figure S13: Photographic images of non-crosslinked PGl fibres (a), fully crosslinked PGl/HM-bisTAD fibres (b) and fully crosslinked PGl/MDP-bisTAD fibres (c).
